# Element-Based Predictive Modeling of Hydrothermal Liquefaction Bioproducts Derived from Corn Stover

**DOI:** 10.1021/acssuschemeng.5c09967

**Published:** 2025-12-26

**Authors:** Isamu Umeda, Meicen Liu, Yi Zheng, Jiefu Wang, Zhiwu Wang, Sandeep Kumar

**Affiliations:** † Department of Civil and Environmental Engineering, 250778 Old Dominion University, Norfolk, Virginia 23529, United States; ‡ Department of Grain Science and Industry, 5308 Kansas State University, 1980 Kimball Avenue, Manhattan, Kansas 66506, United States; § Department of Biological Systems Engineering, Virginia Polytechnic Institute and State University, 1230 Washington St. SW, Blacksburg, Virginia 24060, United States

**Keywords:** lignocellulosic biomass, biocrude, predictive modeling, correlation, kinetic model, induction heating

## Abstract

The hydrothermal liquefaction (HTL) process offers an energetic advantage over pyrolysis because it does not require prior drying of the biomass feedstock. However, there are significant challenges in simultaneously estimating both the yields and characteristics of products from the HTL of biomass with theoretical support. This study developed a unique element-based kinetic model to predict the yields, higher heating values, and fuel characteristics of solid residue and heavy bio-oil, based on the temperature, residence time, solid loading, and elemental composition (C, H, N, and O) of corn stover. Furthermore, the model predicted the weights of dissolved carbon and nitrogen in the aqueous phase. HTL experiments were conducted using corn stover at temperatures ranging from 250 to 350 °C for residence times between 5 and 60 min. The resulting solid and liquid products were analyzed for the elemental composition and ash content. The experimental data and MATLAB program were used to predict the products. The fuel characteristics derived from predicted elemental weight data of solid residues followed the trend line of the observed data on the van Krevelen diagram. In those of heavy bio-oil, the H/C atomic ratio of the average predicted data matched the one calculated from the observed data. Additionally, power function relationships between the amounts of corn stover and obtained product fractions were identified under identical temperature and residence time conditions by varying solid loading, providing insights into the partial nonlinear behavior of the reaction system.

## Graphical abstract

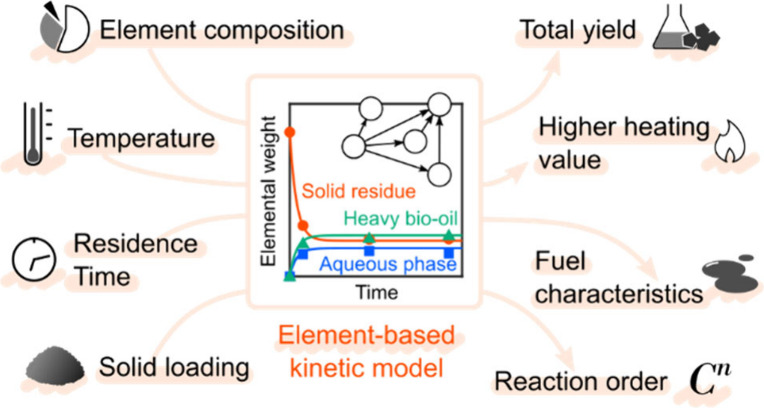

## 1 Introduction

Biofuels are viable alternatives to conventional petroleum-based fuels and are crucial for a sustainable future due to their renewability and environmental benefits. Significant progress has been made in the development of biofuels, such as biodiesel and bioethanol, which have already been commercialized. However, given that the modern lifestyle still heavily relies on petroleum-based fuels, a substantial increase in biofuel production is anticipated to meet future demands.

Bio-oil or biocrude, which can be catalytically upgraded into sustainable aviation fuel (SAF) or other drop-in transportation fuels, is thermochemically produced from lignocellulosic materials through processes, such as pyrolysis and hydrothermal liquefaction (HTL). One of the key advantages of HTL is its versatility to process a broad range of feedstock, including terrestrial biomass, aquatic biomass, and any kind of organic wastes (e.g., municipal solid waste and manure). By fully utilizing lignocellulosic materials, biocrude has the potential to meet the demand for biofuels without competing with food resources, such as vegetable oils and starch. In particular, the HTL process offers an energetic advantage over pyrolysis as it does not require the biomass feedstock to be dried beforehand.

Utilizing crop residue, which is a lignocellulosic material, creates a win-win situation for both farmers and the alternative energy sector. Corn stover (CS), one of the most abundant crop residues in the United States, is the nonedible residue left in fields after corn harvesting. In 2012, approximately 300 million tons of stover were produced across the United States,^1^ which include leaves, stalks, husks, and cobs.^2^ U.S. corn production as feed grain has generally increased despite year-to-year fluctuations, and production in the 2025/26 marketing year is projected to be the largest since 1978.^3,4^ These data imply an increase in corn stover production, exceeding that of 2012. Recent studies have explored the cohydrothermal liquefaction (co-HTL) of CS with other biomass materials (e.g., manure) to improve the quality and yield of biocrude.^5^

There are several types of predictive models: component additivity models, kinetic models, and machine learning models.^6^ Component additivity models solve regression models that mainly include variables representing biochemical components, such as carbohydrates, lipids, and proteins. Kinetic models numerically solve differential equations for the concentrations of fractions, which are constructed based on assumed reaction pathways. Component additivity models and kinetic models provide a framework to associate independent variables with dependent variables through explicit equations. This approach is effective when appropriate model equations can be developed based on well-established theories or relationships underlying the phenomena. Machine learning models allow for the precise prediction of bioproducts. For most nonlinear machine learning models, the relationships between independent and dependent variables cannot be expressed as explicit equations, although partial dependence plots (PDPs) and SHAP values can illustrate how each independent variable influences the outcomes. Since the HTL process involves thermochemical reactions, it is typically modeled using a kinetic framework. In such models, all materials participating in the reactions are represented within a reaction network, which defines the reaction pathways.

The reaction rates are described by ordinary differential equations (ODEs), which establish relationships among time, material concentrations, and reaction rate constants along the reaction pathway. The reaction rate constant is further expressed as a function of temperature by using the Arrhenius equation. By integration of the ODEs with the Arrhenius equation, the yield can ultimately be expressed as a function of temperature, residence time, and concentration. In practice, this system is often numerically solved by using experimental data. This modeling approach has been widely applied in research on HTL processes involving microalgae,^7–11^ sewage,^8^ and lignocellulosic biomass.^8,12^ Under HTL or pyrolysis conditions, these materials undergo reactions that produce various fractions, as dictated by the chemical reaction network.

In many of these studies, a pseudo-first-order reaction model has been adopted, assuming that water is present in sufficient quantities to react with the feedstock. According to this assumption, the mass fractions of the resulting bioproducts are functions solely of temperature and residence time independent of the initial feedstock quantity. Consequently, these studies often used a fixed feedstock amount, and few have investigated the impact of biomass loading on bioproduct yields due to the prevalence of the pseudo-first-order reaction assumption. On the other hand, Nava-Bravo et al. reported that 10 wt % of CS was more favorable than 20 wt % for achieving a higher biocrude yield showing a difference of approximately 6.9 wt % at 300 °C in yield.^13^ This clearly indicates that the biocrude yield is influenced by the feedstock loading and that not all reaction pathways can be assumed to follow pseudo-first-order kinetics. Therefore, it is important to consider how the initial amount of feedstock affects the product fraction yields, as investigated in the present work.

Regarding the unit of measurement for fractions, kinetic models commonly use the molar concentration. This molecule-based approach is an appropriate method for representing kinetics. In practice, however, mass concentration instead of molar concentration has also been used in kinetic models.^14^ To implement this conversion, the average molecular mass of each fraction must be determined in advance. For example, in the case of a reaction pathway $A\overset{k}{\to }B$ , the rate equation based on molar concentration is expressed as

1 $$\frac{d\left[B\right]}{dt}=k\left[A\right]$$

which can be converted to a mass-based equation as follows:

2 $$\frac{dw_{B}}{dt}=k\frac{M_{B}}{M_{A}}w_{A}$$

where [X], *w*_X_, *M*_X_, and *k* represent the molar concentration, weight, average molecular mass of fraction X, and the reaction rate constant, respectively. To minimize the number of variables and simplify the model, mass concentrations of materials were used instead of molar concentrations, as shown below:

$$\frac{dw_{B}}{dt}=k^{\prime }w_{A}$$ 3

Using mass-based fractions in the kinetic models demonstrates the approach’s practicality and effectiveness. Considering that the mass of starting materials and product fractions often changes exponentially over time, similar to molar concentration-based trends, mass-based kinetic models remain effective for describing complex weight changes. Most studies utilizing kinetic models have focused on predicting the yields of biocrude or solid residues.

A kinetic model is extended by incorporating elemental weights, an element-based kinetic model can be expressed as follows:

4 $$\frac{d{\left[B\right]}_{E}}{dt}=k_{E}{\left[A\right]}_{E}\Rightarrow \frac{d}{dt}w_{B,E}=k_{E}w_{A,E}$$

where [X]_E_, *M*_E_, and *k*_E_ represent the molar concentration, average molecular mass of fraction *X* regarding element E, and the reaction rate constant for element E, respectively. E can represent elements in the fractions, such as carbon (C), hydrogen (H), nitrogen (N), or oxygen (O). The advantage of this element-based kinetic model is that handling mass-based ODEs is mathematically equivalent to molar concentration-based models, as the atomic mass of the element cancels out in the equations. Thus, this element-based kinetic model is expected to perform similarly to the original molar concentration-based kinetic model. Moreover, since the elemental weights of fractions can be separately measured using ultimate analysis, this approach allows not only the prediction of each fraction’s total weight but also the calculation of HHVs and insight into fuel characteristics, such as H/C and O/C atomic ratios. Furthermore, because neither average molecular weight nor corresponding stoichiometric coefficients are included in the ODEs, this approach reduces the number of variables that would otherwise be underdetermined or difficult to initialize and that could complicate the numerical solution of the ODEs. Nevertheless, little information is available about the application of this approach in predicting or correlating the outcomes of the HTL process.

Rapid heating of the reactor system is advantageous for maximizing the biocrude yield. For instance, the yield of biocrude derived from algal biomass at 300–400 °C was maximized within 3–7 min, surpassing the maximum yield produced at 250 °C.^7^ Minimizing the preheating time in HTL is essential for facilitating accurate kinetic analysis at the target temperature.^15^ To achieve such rapid heating, Hirayama implemented fast HTL employing an induction heating unit and a 280 mL reactor which could achieve a heating rate of approximately 100 °C·min^–1^.^16^

This study aims to develop and validate an element-based kinetic model to predict the yields and characteristics of bioproducts derived from CS. The novelty of this approach lies in its ability to use elemental balances to not only predict product yields (SR, AP solutes, and HBO) but also estimate their HHV and fuel characteristics through H/C and O/C ratios. By explicitly incorporating solid loading as a variable, the work provides new insights into the effect of feedstock mass on product distribution and tests the validity of the pseudo-first-order assumption. Induction heating was employed to minimize the preheating duration, enabling a clearer interpretation of temperature- and time-dependent reactions.

## 2 Experimental Section

### 2.1 Materials

The elemental compositions (dry basis) of CS used as feedstock for HTL in this study include 55.58 wt % C, 6.50 wt % H, 1.09 wt % N, and 5.18 wt % ash, with the remainder being oxygen by difference. The selected particle size of CS was such that it passed through a 12.7 mm sieve and was retained on a 2 mm sieve. Prior to HTL treatment, the CS was dried in an oven at 105 °C for 16 h. Acetone (≥99.5%, Thermo Scientific, USA) was used as a solvent for the collection of HBO from the HTL products after cooling the reactor to room temperature. Filter papers (11 μm, Whatman 1, Cytiva, USA) were employed to separate the HTL mixture. After being wetted with deionized water once, the filter papers were dried at 105 °C and weighed using a moisture analyzer (IR-35, Denver Instrument, USA).

### 2.2 HTL Treatment

The dried CS (5, 10, and 15 g) and 100 g of deionized water were placed into a 280 mL stainless steel batch reactor (GC-3, High Pressure Equipment Co., USA). Two thermocouples were attached to the reactor vessel to monitor the central external surface temperature and the outer surface temperature of the vessel. The temperatures measured by these thermocouples were regarded as the inner and outer temperatures of the vessel, respectively. Figure 1 illustrates the HTL treatment system with its induction heating unit. To quickly heat the vessel, an induction heating unit (HI-HEATER4020, DHF, Japan) equipped with a PID feedback controller was used. As the inner temperature approached the target temperature (250, 300, and 350 °C), the heating rate decelerated due to the PID feedback control. If the residence time starts when the inner temperature exactly reaches the target temperature, then the duration to reach this point could be significantly long, potentially affecting the treatment extension. Therefore, the residence time initiation temperature was defined as the moment when the inner temperature reached a value 5 °C below the target temperature, specifically 245, 295, and 345 °C. Heating rates were calculated by dividing the temperature increase by the time required to reach the target temperature, resulting in values ranging from 28 to 98 °C/min, with an average of 65 °C/min. The relationships among these variables are represented in Figure 2. Once the inner temperature reached the target temperature, it was maintained for the predetermined residence times (5, 30, and 60 min), after which the induction heating was turned off. The vessel was then removed from the heating coil box and cooled with running tap water for approximately 5 min. This procedure was repeated 81 times under varying conditions, including repetitions (3 temperature levels × 3 residence time levels × 3 solid loading levels × 3 replicates per condition).

**Figure 1:**
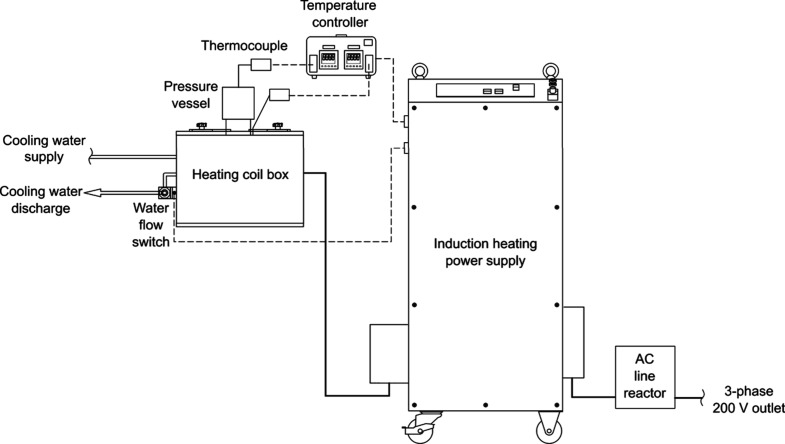
Inductively heated HTL system.

**Figure 2:**
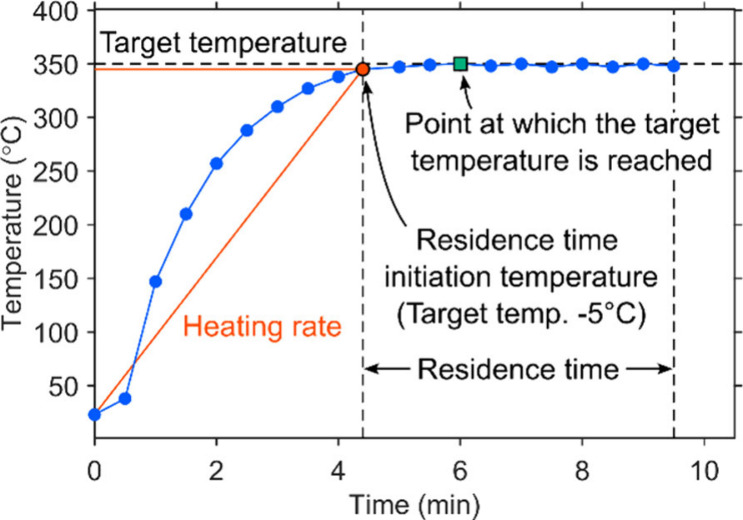
Schematic representation of how the target temperature, residence time initiation temperature, heating rate, and residence time are related in a temporal temperature profile. The green square shows the point when the temperature reached the target temperature.

### 2.3 Post-Treatment

Scheme 1 illustrates an overview of the sequence for collecting, separating, and extracting samples. After each HTL treatment, the reactor was opened and the mixture contents were poured into a beaker. To recover as much of the mixture as possible, the inner surface of the vessel was scraped with a spatula and washed twice with 25 mL portions of acetone (50 mL in total). The acetone washes were collected in a separate beaker. The vacuum filtration was conducted using a Büchner funnel, suction bottle, and vacuum pump. The mixture was then separated into a solid mixture and AP through suction filtration. The AP collected was weighed, and its pH was measured. The solid mixture was removed from the filter paper, combined with the acetone washings, and mixed to extract HBO. The acetone–solid mixture was then poured back onto the same filter paper and separated into the acetone washings and solid mixture through filtration. The solid mixture was further rinsed twice with an additional 25 mL of acetone, filtered again using the same filter paper, and collected as the SR. In total, 100 mL of acetone was used. The SR, along with the filter paper, was dried and weighed by using the moisture analyzer. When some of the SR ignited during drying, the SR were instead dried in an oven at 105 °C for at least 24 h before weighing. The weight difference between the dried residue and the original filter paper was recorded, and the total SR weight was obtained by summing the results of three trials.

**Scheme 1:**
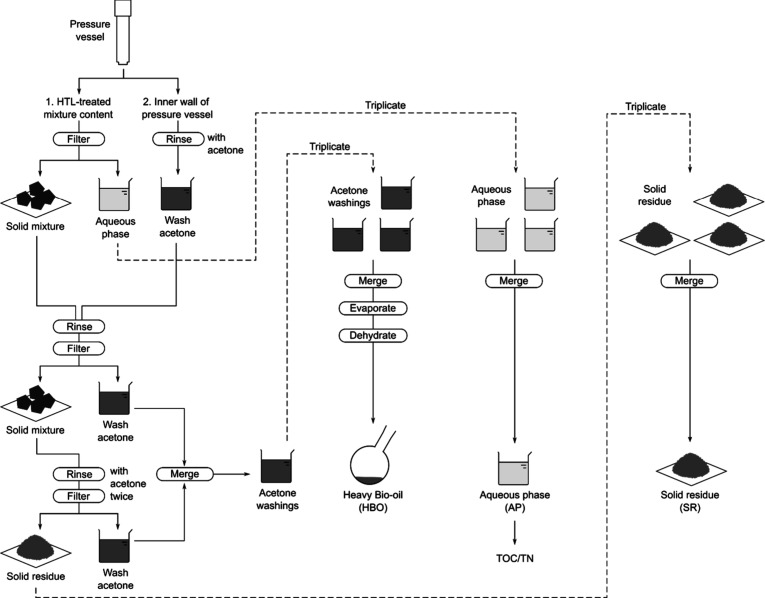
Overview of the Post-Treatment

The acetone washings obtained from three replicates of HTL treatment were combined, transferred to a preweighed boiling flask, and evaporated using a rotary evaporator under vacuum conditions by a recirculating water aspirator at 65 °C for 30 min. As the acetone washings often contained water, the evaporation process resulted in the separation of the water and HBO fractions. The water fraction remaining in the flask was discarded, and the HBO was dehydrated in an oven at 65 °C for 30 min. The flask was then cooled to room temperature and weighed to calculate the final amount of HBO by subtracting the original weight of the flask. The AP obtained from triplicate HTL treatments was merged into a single sample and weighed. For the analysis of total organic carbon (TOC) and total nitrogen (TN) in the AP, 15 mL of the AP was taken and diluted 40-fold with Milli-Q water. Here, the term AP solutes is defined as soluble carbon and nitrogen, which were quantified as TOC and TN.

### 2.4 Product Analysis

The pH of the AP was measured using a multimeter (ORION 5 STAR, Thermo Scientific, USA) equipped with a pH probe (ORION 8102BNUWP, Thermo Scientific, USA). The TOC and TN of the AP were analyzed on a TOC analyzer (TOC-V CSN, SHIMADZU, Japan) equipped with a total nitrogen measuring unit (TNM-1, SHIMADZU, Japan) and an autosampler (ASI-V, SHIMADZU, Japan). CHNS analysis of HBO and SR was performed using an organic elemental analyzer (Flash 2000, Thermo Scientific, USA) with the BBOT standard (CE Elantech, USA) for calibration. For the measurement of ash content in HBO and SR, each sample was placed in a preweighed crucible and combusted at 575 °C for 16 ± 8 h in a furnace. After combustion, the samples were allowed to cool in a desiccator for 1 h and then weighed. The net weights of the raw fraction and its ash were calculated by subtracting the weight of the crucible, allowing the ash content to be determined as a percentage. The oxygen content in HBO and SR was calculated using the results of the ash content and CHNS analyses with the following equation: O% = 100% – (C% + H% + N% + Ash%). The HHV was calculated using Dulong’s formula (eq 5):^17^

5 HHV(MJ/kg)=0.335(C%)+1.423(H%)−0.154(O%)−0.145(N%)

### 2.5 Kinetic Model and Prediction

The kinetic model illustrated in Figure 3 was adopted. A chemical reaction network was hypothesized, where the circled nodes represent the starting feedstock (CS) and the fractions obtained in the HTL process. The arrows between the nodes indicate reaction pathways, with *k*_*i*_ on each arrow denoting the corresponding reaction rate constant. All reaction pathways were assumed to follow pseudo-first-order kinetics, as described by the system of ordinary differential equations (ODEs) in eqs 6–10.

6 $$\frac{dy_{\mathrm{CS},E}}{dt}=\left(-k_{1,E}-k_{2,E}-k_{3,E}-k_{6,E}\right)\cdot y_{\mathrm{CS},E}$$

7 $$\frac{dy_{\mathrm{HC},E}}{dt}=k_{1,E}\cdot y_{\mathrm{CS},E}+k_{4,E}\cdot y_{\mathrm{AP},E}+k_{5,E}\cdot y_{\mathrm{HBO},E}$$

8 $$\frac{dy_{\mathrm{AP},E}}{dt}=k_{2,E}\cdot y_{\mathrm{CS},E}-k_{4,E}\cdot y_{\mathrm{AP},E}$$

9 $$\frac{dy_{\mathrm{HBO},E}}{dt}=k_{3,E}\cdot y_{\mathrm{CS},E}-k_{5,E}\cdot y_{\mathrm{HBO},E}$$

10 $$\frac{dy_{\mathrm{Gas},E}}{dt}=k_{6,E}\cdot y_{\mathrm{CS},E}$$

where the subscript E denotes the element (C, H, N, or O), indicating that each ODE is element-specific. *y*_CS, E_, *y*_HC, E_, *y*_HBO, E_, *y*_AP, E_, and y_Gas, E_ represent a given element-based weight (g) of each fraction.

**Figure 3:**
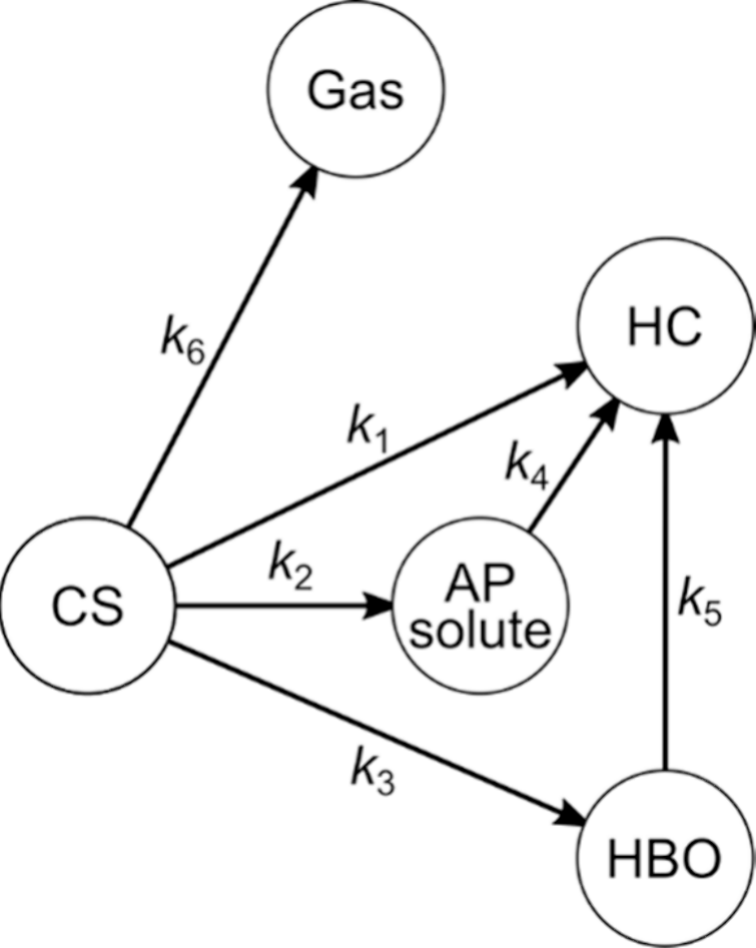
Assumed chemical reaction network.

The reaction pathways denoted by *k*_1_, *k*_2_, *k*_3_, and *k*_6_ describe the direct conversion of CS to other fractions. Additionally, pathways *k*_4_ and *k*_5_ represent the conversion of AP and HBO into HC. These reaction pathways capture the increase in HC after the decomposition of CS. Kinetic models used for the HTL of microalgae or its model substances frequently include solid starting materials.^7–12,18–21^ In some studies, HC was either omitted from existing kinetic models or not distinguished from the starting materials (i.e., the system included a reversible reaction involving the solid starting material). It was reported that HC was produced during the HTL of CS from 210 to 375 °C.^22^ A kinetic model incorporating a reaction network that includes a solid fraction in addition to the feedstock was established.^8^ In the current research, the HC fraction was explicitly included in the model alongside CS.

### 2.6 Data Analysis

The data analysis was performed using MATLAB R2024a. Curve fittings to the observed data were conducted to optimize the pre-exponential factor (*A*_E_) and activation energy (*E*_a, E_) in the Arrhenius equation, *k* = *A*_E_·exp­(−*E*_a, E_/*RT*), where *R* is the gas constant (8.314 J·mol^–1^·K^–1^) and *T* is the temperature in Kelvin. Since SR was the total weight of the untreated CS and HC after HTL treatments, predicted SR was calculated by merging separately predicted CS and HC (*y*_SR, E_ = *y*_CS, E_ + *y*_HC, E_). The calculation primarily utilized the *lsqcurvefit* function and the ODE solver *ode15s*. The observed data and initial values of *A*_E_ and *E*_a, E_ were passed to a function incorporating *ode15s* and the reaction rate of the ODEs (eqs 6–10). By numerically solving the ODEs with the input data, predicted element-based yields were simulated. The *lsqcurvefit* function, a least-squares fitting algorithm, iteratively adjusted *A*_E_ and *E*_a, E_ to minimize the sum of squared residuals between the predicted and observed yields. This process ultimately provided the optimized values of *A*_E_ and *E*_a, E_, as well as the element-based weights over time for each condition.

The differences between the observed data and the predicted data were quantified using the root mean squared error (RMSE), combined RMSE (RMSE_2_), and relative error (RE) as defined in eqs 11–13, respectively:

$$\mathrm{RMSE}=\sqrt{\frac{1}{n}\overset{n}{\underset{i=1}{\sum }}{\left(y_{i}-{ŷ}_{i}\right)}^{2}}$$ 11

$${\mathrm{RMSE}}_{2}=\sqrt{\frac{1}{2n}\overset{n}{\underset{i=1}{\sum }}\left[{\left(y_{1,i}-{ŷ}_{1,i}\right)}^{2}+{\left(y_{2,i}-{ŷ}_{2,i}\right)}^{2}\right]}$$ 12

13 $$\mathrm{RE}=\frac{{ŷ}_{i}}{y_{i}}-1$$

where *n* is the number of actual data points, *y*_*i*_ and *ŷ*_*i*_ represent the observed and predicted values, and the subscripts 1 and 2 refer to the first and second components.

To evaluate the fuel characteristics of the SR and HBO obtained, van Krevelen diagrams were used. These diagrams illustrate the fuel characteristics of carbonaceous materials by plotting the H/C atomic ratio against the O/C atomic ratio. Using the measured weights of carbon, hydrogen, and oxygen for each fraction, the H/C and the O/C atomic ratios were calculated.

## 3 Results and Discussion

### 3.1 Element-Based Kinetic Analysis

Table 1 lists the values of *A*_E_ and *E*_a, E_ for each element obtained from the simulation, which ranged from 1.00 s^–1^ to 2.86 × 10^6^ s^–1^ and from 29.6 kJ·mol^–1^ to 259 kJ·mol^–1^, respectively. All element-based *E*_a, E_ for *k*_4_, which represents the pathway from AP solute to HC, was high compared with the other pathways. Given that particles appeared from AP even at refrigerator temperature,^23^ such high activation energies appear counterintuitive. This discrepancy arises because these are apparent activation energies. At higher temperatures, AP contains a higher concentration of solutes that tend to precipitate upon cooling, leading to the formation of a larger quantity of solid particles. Therefore, the process described by the pathway from the AP solute to the HC is not considered to be directly governed by temperature.

**Table 1:** Element-Based Pre-Exponential Factors and Activation Energies in Fractions^a^

	*A*_E_ (s^–1^)					
element	*k*_1_	*k*_2_	*k*_3_	*k*_4_	*k*_5_	*k*_6_
C	3.10 × 10^2^	1.07 × 10^5^	1.88 × 10^6^	8.72 × 10^4^	7.00 × 10^4^	3.67 × 10^5^
H	1.16	2.34 × 10^5^	2.86 × 10^6^	1.13 × 10^5^	9.53 × 10^5^	3.57 × 10^5^
N	8.68 × 10^2^	2.85 × 10^4^	2.85 × 10^6^	2.90 × 10^2^	2.94 × 10^4^	7.63 × 10^4^
O	1.00	1.41 × 10^6^	2.67 × 10^6^	3.69 × 10^5^	6.62 × 10^5^	1.50 × 10^6^

	*E*_a, E_ (kJ·mol^–1^)					
element	*k*_1_	*k*_2_	*k*_3_	*k*_4_	*k*_5_	*k*_6_
C	52.9	79.3	90.1	259	157	83.2
H	31.3	84.9	94.4	240	123	82.2
N	29.6	46.5	63.7	214	104	43.5
O	32.3	91.7	97.4	243	117	91.8

a *A*_E_ (element-based pre-exponential factor) and *E*_a, E_ (element-based activation energy) in the Arrhenius equation for each assumed reaction pathway (*k*_*i*_).

### 3.2 Weights of SR, HBO, and AP Solute

Figures 4a, 5a, 6a, and 7a show the changes in element-based carbon weights of the fractions over the reaction period. The markers and lines represent the observed and predicted data, respectively. Figures 4b, 5b, 6b, and 7b present parity plots of the element-based weights, where the *x*-axis indicates the weight from the experimental data, and the *y*-axis indicates the corresponding predicted weight.

**Figure 4:**
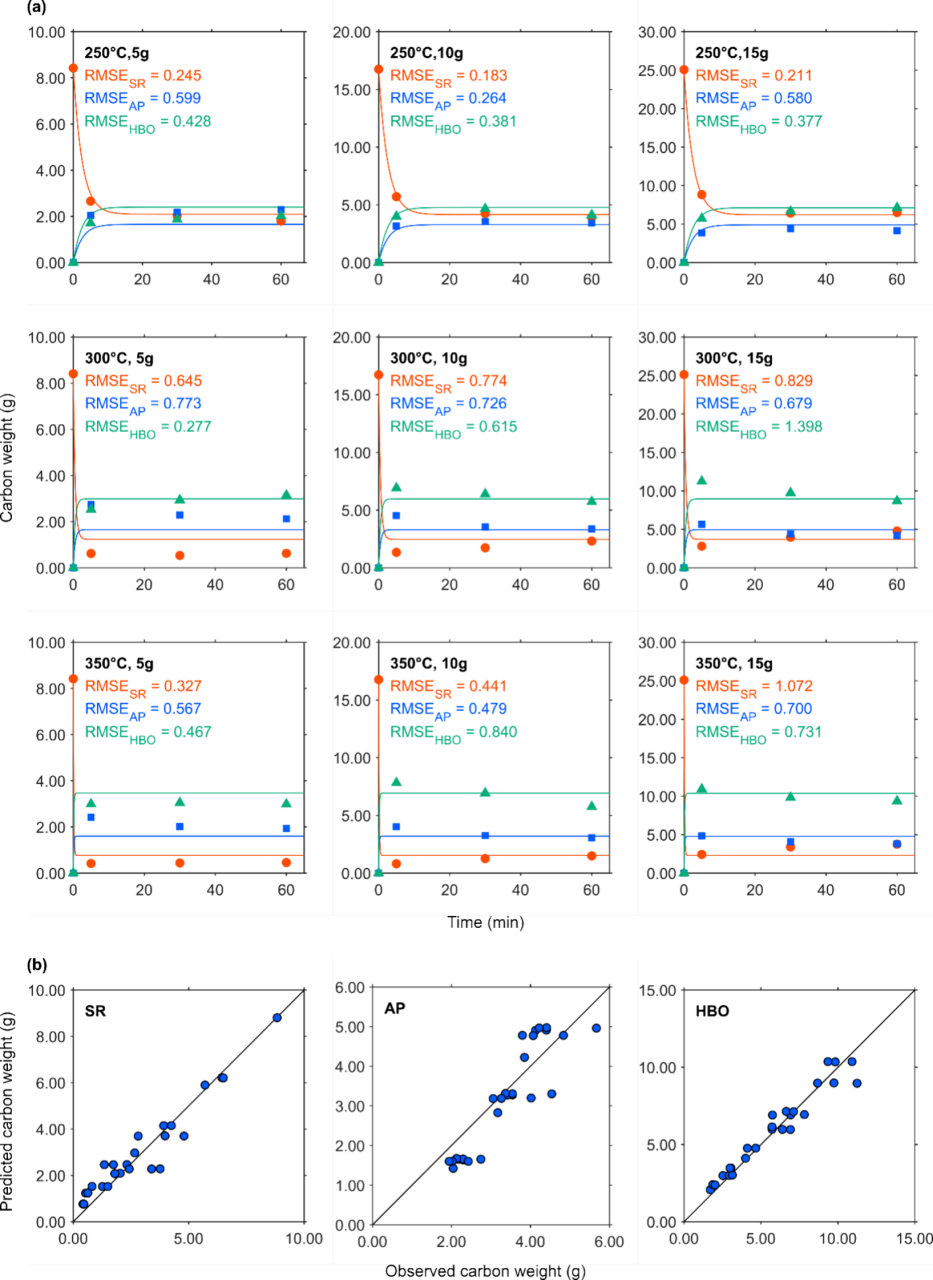
Actual and simulated carbon weight of fractions. (a) ●, ■, and ▲ depict the carbon weight of the solid residue, TOC weight in AP, and carbon weight of HBO, respectively. Lines with the same colors as the markers depict predicted data for each corresponding fraction. (b) Parity plots of SR, TOC in AP, and HBO.

**Figure 5:**
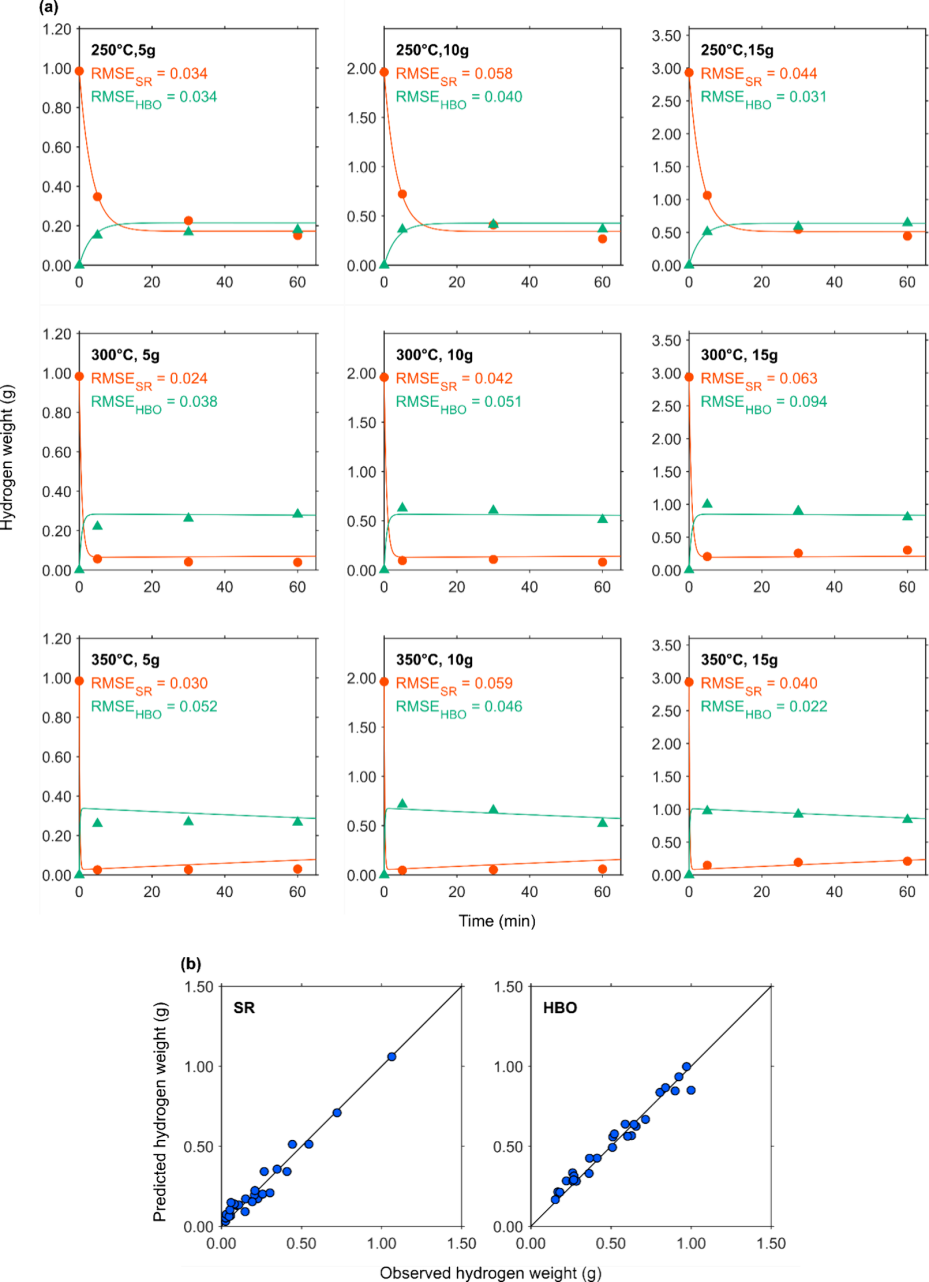
Actual and simulated hydrogen weights of fractions. (a) ● and ▲ depict hydrogen weight of SR and HBO, respectively. Lines with the same colors as the markers depict predicted data for each corresponding fraction. (b) Parity plots for hydrogen weight of SR and HBO.

**Figure 6:**
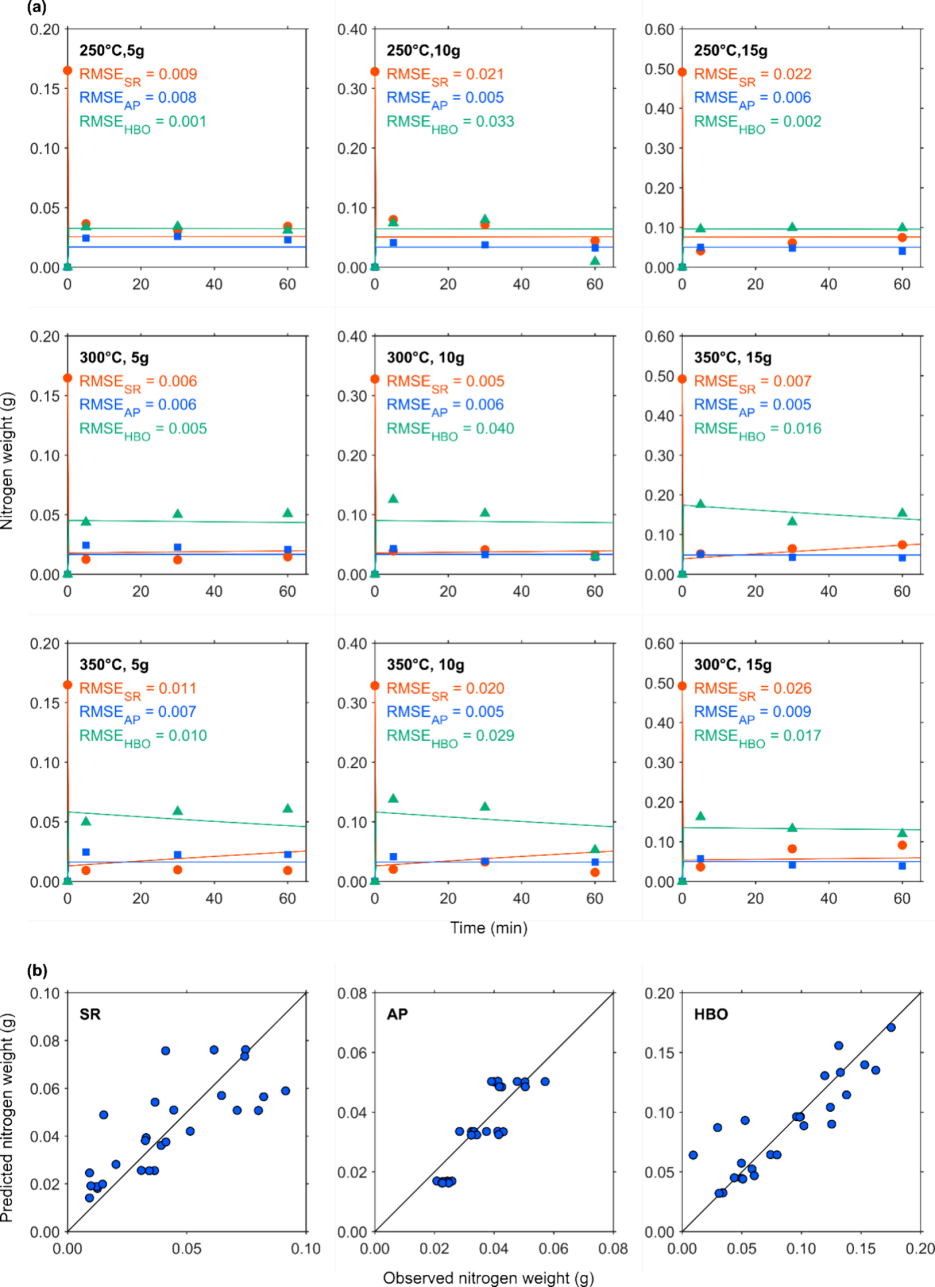
Actual and simulated nitrogen weight of fractions. (a) ●, ■, and ▲ depict the nitrogen weight of SR, the TN weight in AP, and the nitrogen weight of HBO, respectively. Lines with the same colors as the markers depict predicted data for each corresponding fraction. (b) Parity plots of nitrogen weight of SR, TN in AP, and nitrogen weight of HBO.

**Figure 7:**
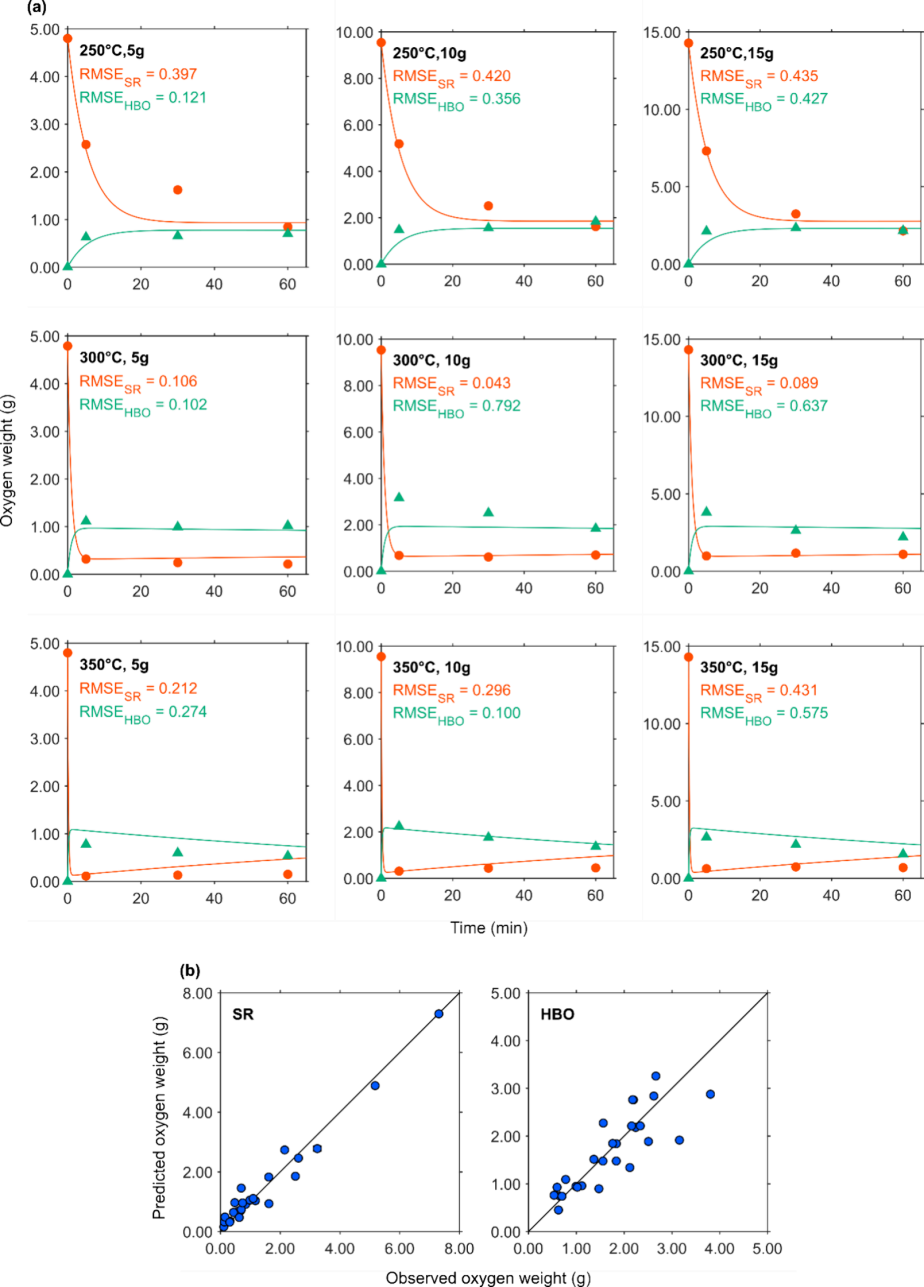
Actual and simulated oxygen weight of fractions over the residence time. (a) ● and ▲ depict the oxygen weight of SR and that of HBO, respectively. Lines with the same colors as the markers depict predicted data for each corresponding fraction. (b) Parity plots of oxygen weight of SR and HBO.

In Figure 4a, the markers represent the carbon weights of the fractions obtained from the HTL of CS over time. At a lower temperature (250 °C), the observed carbon weight of SR initially decreased and then stabilized at approximately 18.9 wt % of the initial CS weight after 60 min. Correspondingly, the carbon weights of AP solute and HBO increased, peaking at around 15 wt % of the initial CS weight. At higher temperatures (300 and 350 °C), the carbon weight of SR rapidly dropped below 10% within the first 5 min and then slightly increased. The carbon weights of the AP solute and HBO reached their maximum values at 5 min but gradually declined thereafter, except for the condition with 5 g of CS at 300 °C.

This trend can be rationalized by considering that the reduction in AP solute and HBO weights may result from their conversion into HC, which subsequently contributes to the regained weight of SR during the HTL process. This observation is consistent with the repolymerization of compounds in the AP solute into carbonaceous materials. In a preliminary test, AP, which was initially a transparent yellow, gradually turned opaque brown over time. This color change was attributed to the formation of fine particles, which could be removed by filtration, restoring the AP to a clear state.

Based on these findings, the modeled reaction network in Figure 3 incorporated pathways from the AP solute to the HC to reflect this process. However, the simulated curves did not fully capture the subtle variations in the observed data. The RMSE values for the carbon weights of SR, HBO, and AP solute reached maximum values of 1.07, 1.40, and 0.77, respectively. Relative to the experimental values, these RMSEs are considered small, suggesting that the simulated curves based on the reaction rate of the ODEs were generally well-fitted to the data, as illustrated in Figure 4a.

Figure 4b presents the parity plots for SR, HBO, and AP solutes, demonstrating the agreement between the simulated and observed values. The markers for SR and HBO closely align with the diagonal, indicating good predictive accuracy. However, the markers for AP solute show a consistent deviation from the diagonal, implying that while the modeled reaction network captured the overall trends, it may still have limitations in accurately describing the carbon distribution in the AP solute.

Figure 5a illustrates the changes in hydrogen weights of the fractions over the residence time. At low temperature of 250 °C, the hydrogen weight of SR continuously decreased, even beyond 30 min. At higher temperatures (300 and 350 °C), the hydrogen weight of SR dropped sharply within the first 5 min and then remained stable. Unlike the carbon weight of SR, its hydrogen weight did not exhibit a notable increase. As shown in Table 2, a slightly higher pH of AP occurred at 350 °C after 30 and 60 min, which is likely that part of the hydrogen in CS was initially converted into soluble organic acids and then polymerized into carbonaceous solids (collected as SR), resulting in a slight increase in the pH and SR weight. The changes in the hydrogen weight of HBO varied depending upon the experimental conditions. Under the conditions of 250 °C/5 g, 300 °C/5 g, and 250 °C/15 g, the hydrogen weight of HBO showed an increasing trend. Conversely, under the conditions of 300 °C/10 g, 300 °C/15 g, 350 °C/10 g, and 350 °C/15 g, it exhibited a decreasing trend. A previous study reported that the concentration of organic acids in the aqueous phase decreases with increasing reaction time when the HTL temperature is at or above 300 °C.^24^ It has been reported that at high temperature (370 °C), the carbon and hydrogen contents in the solid residue increased, accompanied by a decrease in those of the aqueous phase.^22^ These results suggest that certain compounds, including organic acids, might have converted into hydrochar. As with the predictive curves for the carbon weight, the simulated curves for the hydrogen weight aligned well with the experimental data (Figure 5a). The RMSE values were small, and in some cases, the simulations successfully captured subtle variations in the latter part of the time series. Figure 5b presents parity plots for hydrogen weights. The markers for both SR and HBO closely followed the diagonal, indicating that, similar to the case with carbon, the hydrogen weights of SR and HBO were also accurately predicted.

**Table 2:** pH of AP

temperature (°C)	residence time (min)		
(5 g)	5	30	60
250 °C	3.42	3.43	3.41
300 °C	3.25	3.35	3.42
350 °C	3.34	3.59	3.72

temperature (°C)	residence time		
(10 g)	5	30	60
250 °C	3.43	3.41	3.46
300 °C	3.33	3.41	3.40
350 °C	3.39	3.64	3.76

temperature (°C)	residence time		
(15 g)	5	30	60
250 °C	3.47	3.41	3.48
300 °C	3.34	3.43	3.46
350 °C	3.48	3.72	3.78

Figure 6a shows the change in nitrogen weights over residence time. Since nitrogen accounted for only about 1 wt % of the initial CS, all fractions exhibited low nitrogen content. The nitrogen weight of SR decreased, reaching as low as 24.4% of the original CS nitrogen content within the first 5 min.

The simulated curves did not fit the experimental data as closely as they did for carbon and hydrogen weights, and this discrepancy is more clearly illustrated in Figure 6b. The nitrogen weights of SR and HBO showed greater scatter compared with the carbon and hydrogen weights. This increased variability can be attributed to the optimization algorithm used in this study, which minimized the residuals between the observed and predicted values without normalization. As a result, for variables within a smaller range, such as the nitrogen weight of SR, the residuals appeared relatively large, even though the RMSE values remained low. The parity plot of AP solute nitrogen weights shown in Figure 6 exhibited a similar trend to that of AP carbon weights, with the data points offset from the diagonal.

Figure 7a shows the changes in oxygen weights of the fractions over the residence time. At relatively low temperature of 250 °C, the oxygen weight of SR continued to decrease up to 60 min, while the oxygen weight of HBO increased during the first 5 min and then remained stable. At higher temperatures (300 and 350 °C), the oxygen weight of SR decreased sharply within the first 5 min and then stabilized. Similar to the trend observed in the hydrogen weight of SR, a slight increase in oxygen weight was noted after the initial drop.

For HBO, the oxygen weight peaked at around 5 min before gradually declining up to 60 min. The predictive curves for SR and HBO indicate that the steepness of the initial oxygen weight drop increased with the temperature. At 350 °C, the curves showed a slight rise in SR oxygen weight and a modest decline in HBO oxygen weight after the initial decrease. However, these curves deviated slightly from the observed data points.

As shown in Figure 7b, the oxygen weights of HBO exhibited some scatter around the diagonal in the parity plot, similar to the pattern observed for the nitrogen weights. In contrast, the SR oxygen weights displayed less dispersion, with markers aligning more closely along the diagonal, consistent with the trend seen for carbon weights.

The parity plots of the total weight of all elements in the SR, AP solute, and HBO are shown in Figure 8a. The RMSE values for the total weights were 0.73, 0.62, and 1.06 for SR, AP solute, and HBO, respectively. Similar to the parity plots of individual elemental weights presented earlier, the total weight data for SR and HBO aligned closely along the diagonal.

**Figure 8:**
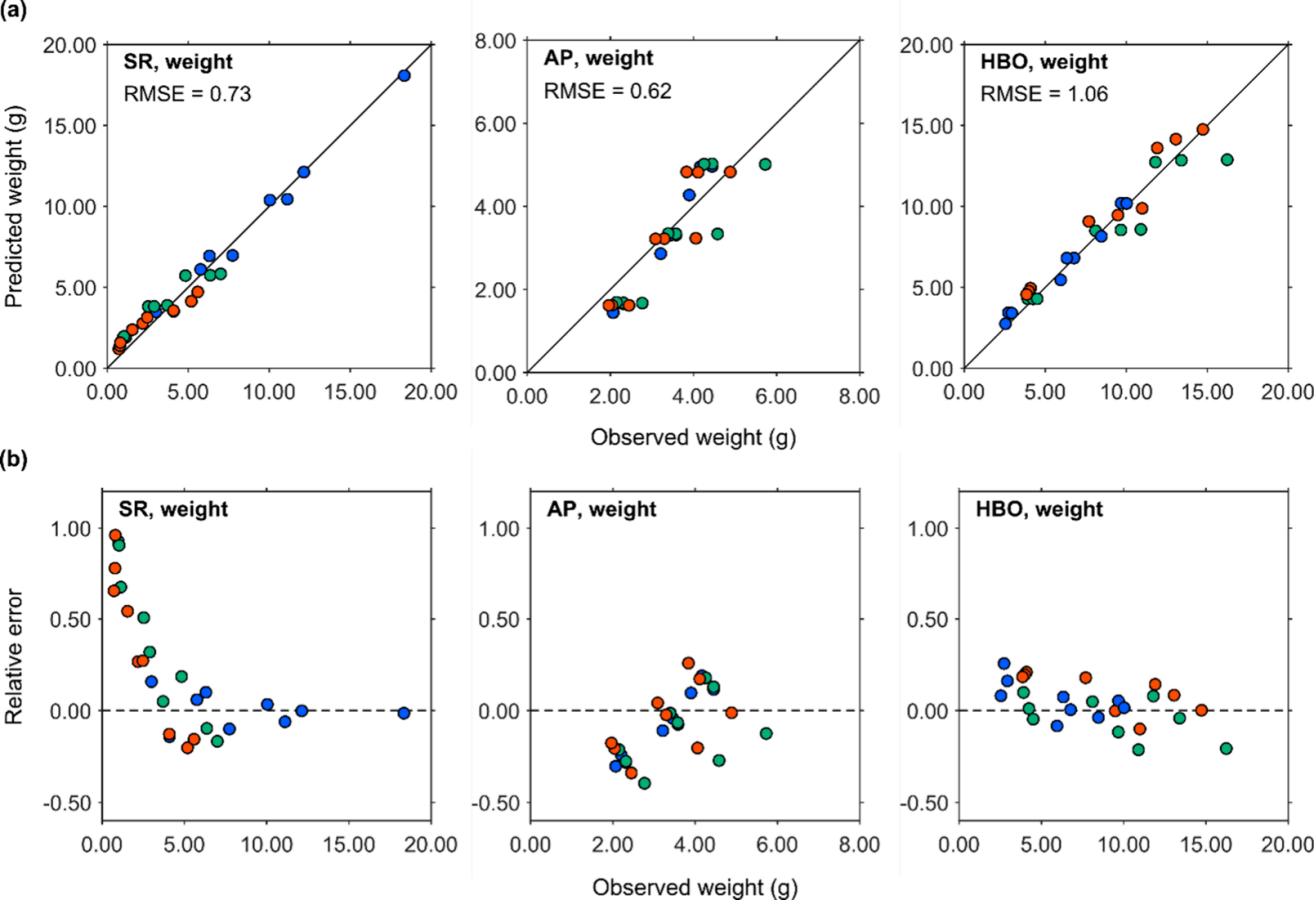
Total weights of SR, AP solute, and HBO. (a) Parity plots. (b) Observed weight vs RE. Blue, green, and red markers indicate points acquired at 250, 300, and 350 °C for HTL treatment, respectively.

As shown in the relative error (RE) of the SR weight in Figure 8b, the error approached 1.00. This can be attributed to the fitting approach used and the small amount of SR. In the current model, the errors between the observed and predicted values were not normalized and were directly used to optimize the fitted curves. This method allows relatively large errors to be accepted at data points where the fraction amounts are small. Consequently, the ratio of predicted to observed values increased, resulting in a large RE gap between the observed and predicted weights.

To improve the accuracy of HHV predictions for SR and HBO, the use of normalized residuals instead of un-normalized residuals may be more effective.

Figures 9a and 9b show the predicted total weights of SR and HBO, respectively, assuming that both are produced from 10 g of CS. According to the simulation results, the condition with 250 °C for 5.0 min yielded the maximum SR weight, resulting in 4.01 g of SR. In contrast, the highest HBO production, 3.27 g, was achieved at 350 °C for 5 min. Since the SR weight includes unreacted CS, it is evident that milder conditions (lower temperature and shorter residence time) result in a larger SR yield. The simulation outcome for SR total weight aligned well with the observed trend.

**Figure 9:**
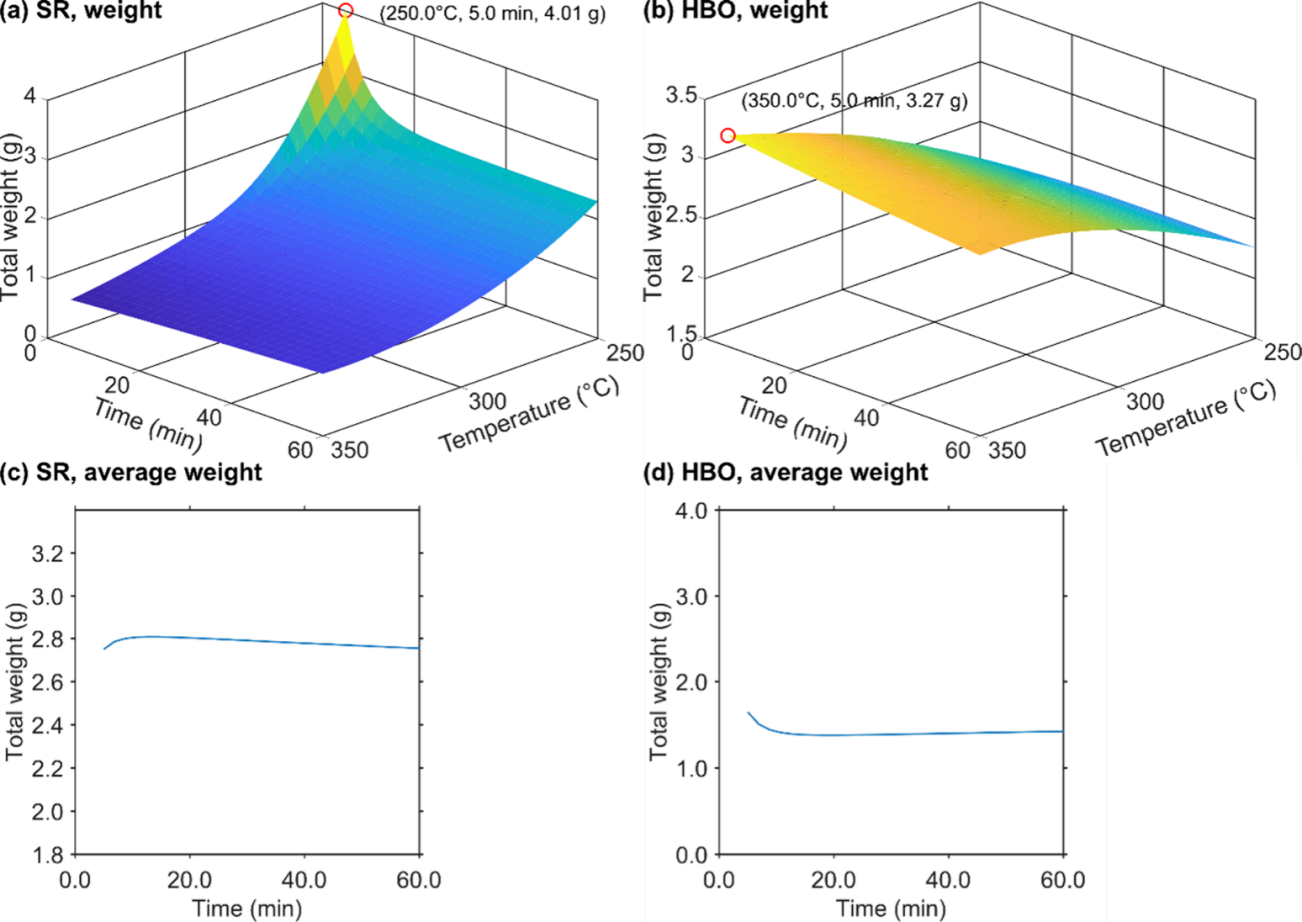
Surface responses of the predicted SR and HBO weights plotted against residence time and temperature at 10 g of CS. (a) SR weight surface, (b) HBO weight surface, (c) change in average total weight projected onto the residence time–total weight plane, and (d) change in average total weight of HBO projected onto the residence time–total weight plane.

The profile of HBO weight resembled that observed for microalgae, where HBO yield increased with residence time at lower temperatures but decreased at higher temperatures.^9^ Figures 9c and 9d present residence time-total weight planes derived by projecting Figures 9a and 9b, respectively, through averaging total weights at each residence time across all temperatures.

Although Figures 9a and 9b show that SR and HBO total weights vary complexly depending on both the residence time and temperature, Figures 9c and 9d reveal that total weights appear nearly constant with respect to the residence time. This indicates that varying residence time alone is insufficient to capture the dynamic behavior arising from simultaneous changes in both residence time and temperature. A similar trend of moderate variation in biocrude yield against residence time has been observed in partial dependence plots from machine-learning-based predictions.

### 3.3 Characteristics of SR and HBO

Figure 10a shows the HHVs of SR and HBO. The average HHVs were 22.8 kJ/g for SR and 21.6 kJ/g for HBO. For SR at 250 °C, the predicted HHVs clustered around 22.7 and 18.3 kJ/g, while the observed values ranged from 17.0 to 24.6 kJ/g. At 300 °C, the predicted HHV was approximately 23.5 kJ/g, with observed values ranging from 18.8 to 26.5 kJ/g. At 350 °C, observed HHVs ranged from 18.7 to 25.8 kJ/g, whereas predicted values ranged from 18.1 to 23.0 kJ/g. The maximum discrepancy between the observed and predicted HHV values was 7.6 kJ/g.

**Figure 10:**
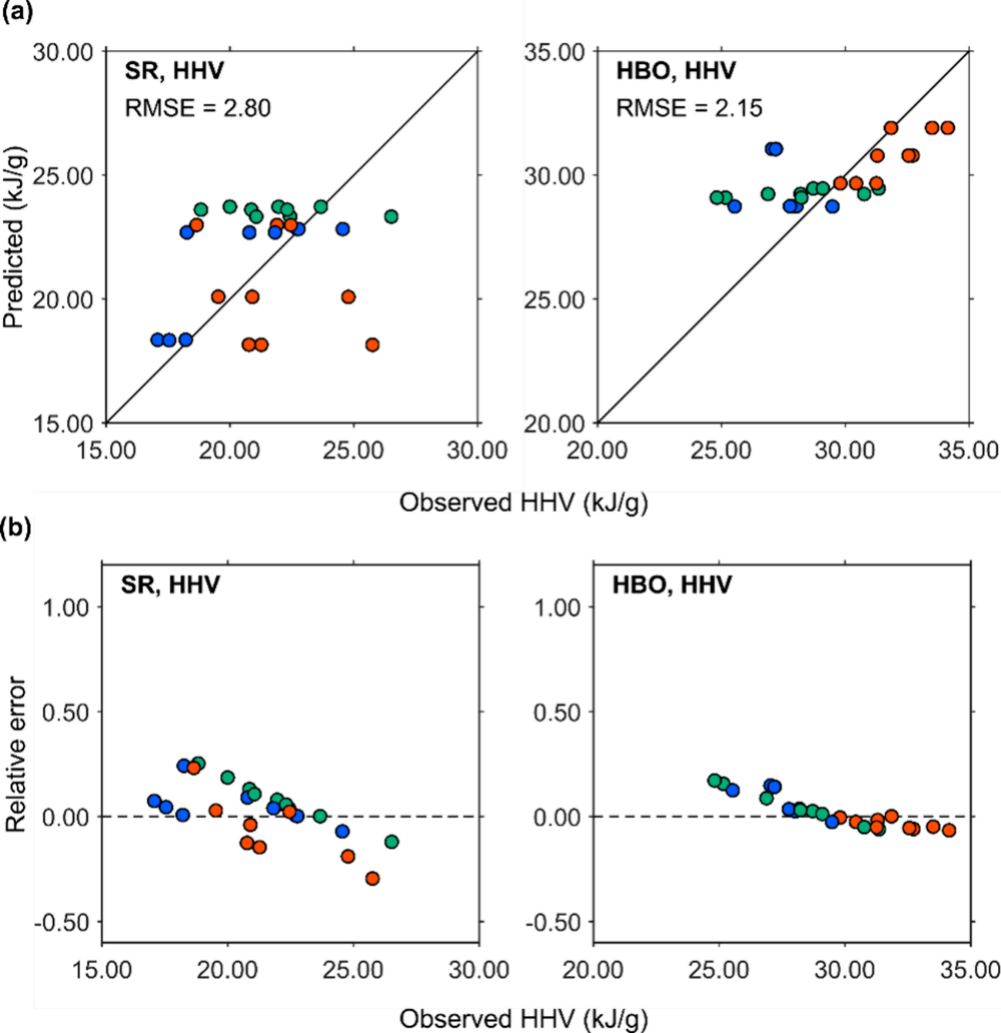
HHV of the SR and HBO. (a) Parity plots and (b) observed HHV vs RE. Blue, green, and red markers indicate points acquired at 250, 300, and 350 °C HTL treatment, respectively.

For HBO at 250 °C, the predicted HHVs were 28.8 and 31.1 kJ/g, while the observed values ranged from 25.5 to 28.8 kJ/g. At 300 °C, observed HHVs ranged from 24.8 to 31.3 kJ/g, with a predicted value of approximately 29.2 kJ/g. At 350 °C, the predicted values were slightly more dispersed (29.7 to 31.9 kJ/g), while the observed values ranged from 29.8 to 34.1 kJ/g.

Compared to the parity plots for weight, the HHV data appeared more scattered, likely due to the relatively narrow range of HHV values. Figure 10b shows the REs of the observed HHVs for SR and HBO. The REs for SR ranged from −29.6% to 25.2%, and those for HBO ranged from −6.6% to 17.2%. These results suggest that the HHVs of both SR and HBO were reasonably well predicted, with particularly narrow REs for HBO at 350 °C, ranging from −6.6% to 0.1%.

Figure 11 presents surface plots of the predicted HHVs of SR and HBO as functions of the residence time and temperature. Within the range of 5–60 min and 250–350 °C, the highest HHV of SR (24.0 MJ/kg) was estimated at 305.2 °C and 6.9 min, while that of HBO (31.9 MJ/kg) was predicted at 350 °C and 60 min.

**Figure 11:**
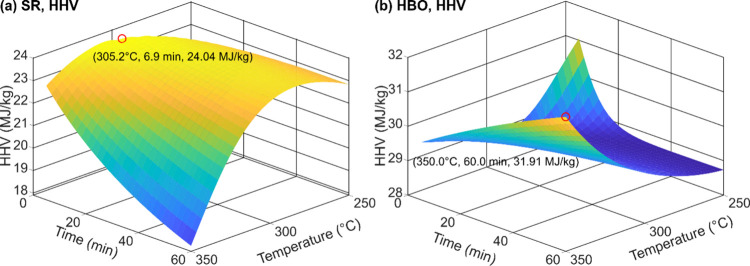
Surface responses of the predicted SR and HBO HHVs plotted against residence time and temperature at 10 g of CS. (a) SR HHV and (b) HBO HHV.

The van Krevelen diagrams of SR and HBO are presented in Figure 12. Blue and red markers represent the observed and predicted data, respectively. Similarly, the blue line indicates the trend line of the observed data, calculated using the least-squares method. The red markers correspond to the data from Figures 4, 5, and 7, which are temporally aligned with the observed data.

**Figure 12:**
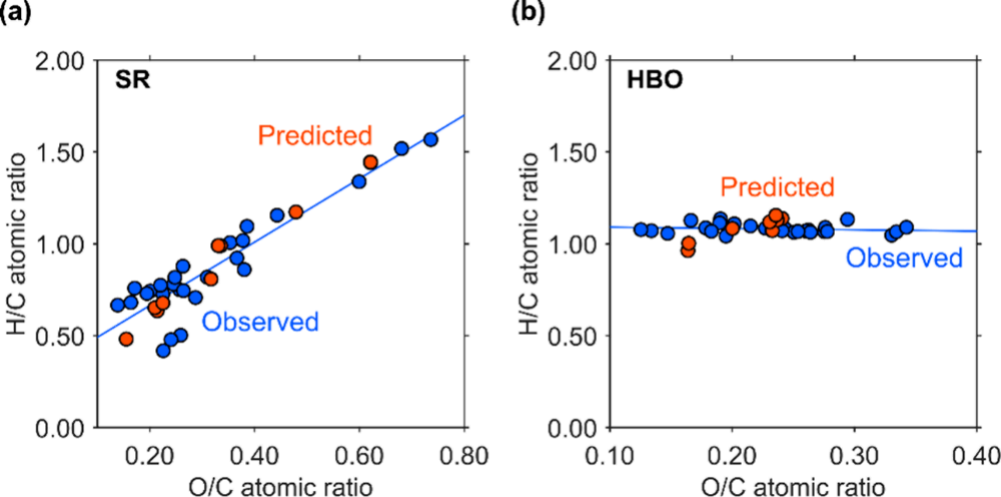
van Krevelen diagrams for the SR and HBO. (a) SR and (b) HBO. Blue and red circle markers refer to observed and predicted data, respectively. The blue lines are trend lines of the observed data.

For SR, the observed data ranged from 0.13 to 0.74 in the O/C atomic ratio and from 0.41 to 1.57 in the H/C atomic ratio. These points formed a diagonal distribution in the van Krevelen diagram, where the H/C atomic ratio increased with an increase in the O/C atomic ratio. The regression line derived from the observed data was expressed as H/C = 1.74 × O/C + 0.32. The predicted SR data ranged from 0.15 to 0.62 for the O/C ratio and from 0.48 to 1.44 for the H/C ratio. As shown in Figure 12a, individual predicted points did not closely match the corresponding observed points (RMSE_2_ = 0.209), while the predicted points followed the overall direction of the observed trend line well (RMSE = 0.061).

For HBO, the observed H/C ratios were clustered around an average of 1.08, ranging from 1.04 to 1.14. The atomic ratios of the two atoms ranged from 0.13 to 0.34, with an average of 0.23, as shown in Figure 12b. The observed trend line was nearly horizontal, indicating that the H/C atomic ratio remained constant despite changes in the O/C ratio. This band was positioned near the upper boundary of the coal region, with part of it extending into the lignite or peat region. The predicted HBO data ranged from 0.96 to 1.16 along the H/C axis, averaging 1.08, and from 0.16 to 0.24 along the O/C axis. The predicted points showed an upward trend in the van Krevelen diagram, diverging slightly from the flat observed trend line. Given that the observed points align nearly horizontally, a horizontal trend can also be identified by averaging the predicted H/C values. As with SR, although individual predicted points did not closely match the corresponding observations (RMSE_2_ = 0.059), the overall deviation from the average observed data was small (RMSE = 0.060).

### 3.4 Impact of Solid Loading on Fraction Weight

An example of the relationship between the amount of CS and the resulting SR obtained from the HTL process is shown in Figure 13a. In some cases, the relationships between CS and SR weights were nonlinear, despite identical treatment conditions for the temperature and residence time. This nonlinearity can be described using the exponent *b* in the power function expression *y*_F_ = *a*·*y*_CS0_^*b*^, where *y*_CS0_ and *y*_F_ represent the initial CS weight and a given fraction weight, respectively.

**Figure 13:**
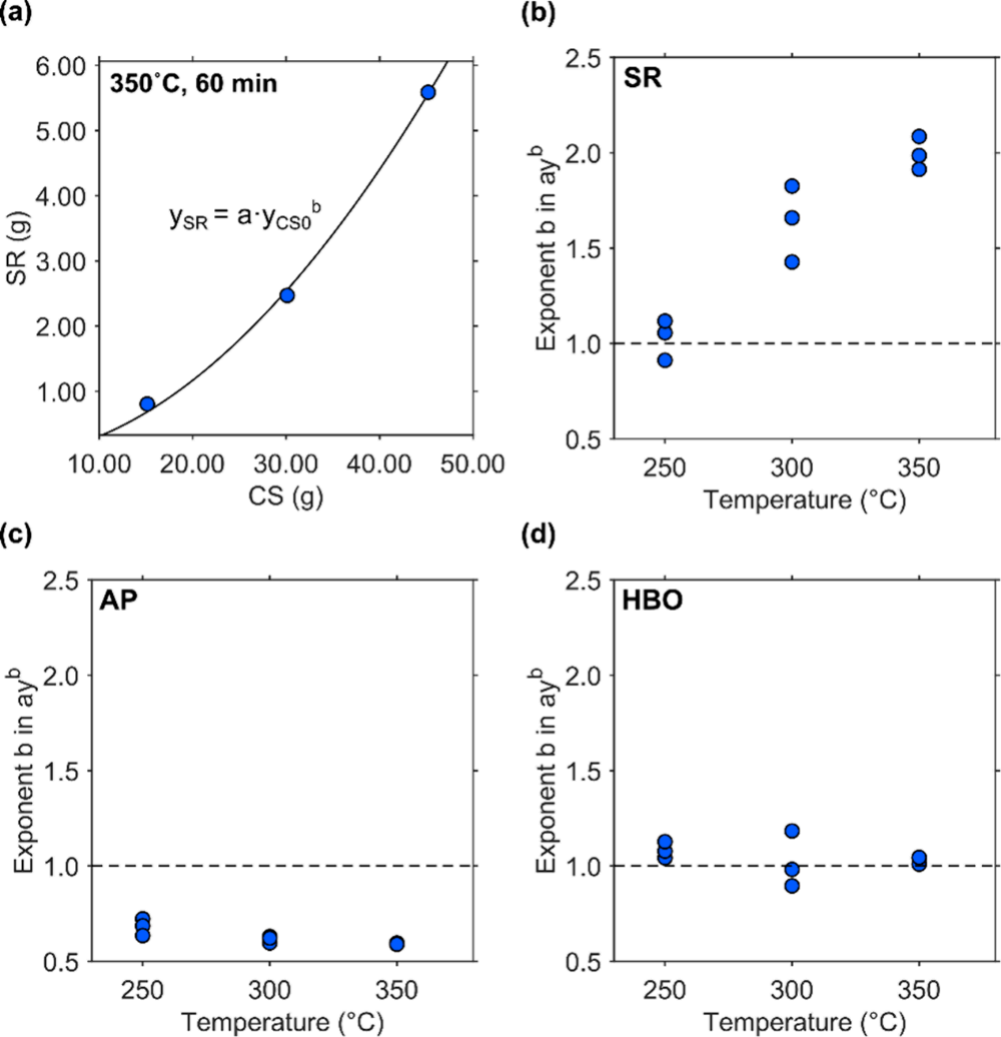
Power functions in relationships among CS, SR, and TOC in AP and HBO weights. (a) Example of relationships between CS and SR weights, (b) relationship between temperature and the exponent that appeared in the relationship of CS and SR weights, (c) relationship between temperature and the exponent that appeared in the relationship of CS and TOC in AP weights, and (d) relationship between temperature and the exponent that appeared in the relationship of CS and HBO weights.

Figure 13b presents the relationship between temperature and the exponent *b* as it appears in the expression *y*_SR_ = *a*·*y*_CS0_^*b*^. The degree of nonlinearity tended to increase as the HTL temperature increased. If the conversion from CS to HC follows a pseudo-first-order reaction, the exponent *b* in the equation *y*_SR_ = *a*·*y*_CS0_^*b*^ should be approximately 1, indicating a proportional relationship between the product and reactant.

The deviation from this expectation suggests potential implications for reaction mechanisms. One possible explanation is that the conversion of CS through HC includes reactions that deviate from pseudo-first-order kinetics. While the prediction of fraction weights based on pseudo-first-order assumptions aligned reasonably well with observed data, it is possible that certain reaction pathways exhibit nonpseudo-first-order behavior.

Figure 13c, which presents analogous diagrams for TOC in AP, shows that the exponents for TOC were less than 1 and decreased with an increase in temperature. This trend contrasts with that of SR, indicating that the TOC-to-CS weight ratio decreased as the initial CS amount increased. This supports the idea that concentrated solutes in AP were converted into SR through non-pseudo-first-order reactions.

Microspheres were produced from the aqueous phase obtained via HTL of switchgrass and corn stover at 200 °C^25^ and from the aqueous phase obtained via HTL of monosaccharides and phenolic compounds at 130 and 170 °C.^26^ Given that the spheres grow through surface reactions between the nuclei and sugar derivatives as well as phenolic compounds, it is likely that the reaction rate increases as the sphere surface area expands. This mechanism would yield an AP solute exponent lower than 1 and an SR exponent greater than 1. In fact, Hietala et al. used a kinetic model that includes a second-order reaction in the polymerization of small polysaccharides into insoluble biochar.^14^

This interpretation also explains the deviation from the diagonal line in the parity plot shown in Figures 4b, 6b, and 8b. At higher concentration of AP solute, AP solutes are consumed faster than predicted by the pseudo-first-order model. At lower concentrations, the opposite occurs, resulting in slower conversion and higher remaining AP solutes compared to the model prediction. This systematic discrepancy produces the observed deviation from the parity line.

Figure 13d illustrates the relationship between the temperature and the exponents derived from the correlation between CS and HBO weights. In this case, the exponents were close to 1, indicating an approximately linear relationship between CS and HBO weights. Of the conditions explored in this study, the dependence of HBO yield on the weight percentage of biomass feedstock, as reported by Nava-Bravo et al.,^13^ was not clearly observed.

### 3.5 Applications, Limitations, and Future Work

In this work, 5 min was used as the minimum residence time. Examination of the predicted elemental weight profiles shows that most curves drop sharply within a very short period and then remain nearly constant. In future work, additional data will be required during the period of rapid reaction progression, which occurs within the first 5 min under higher-temperature conditions.

Application of this model to a lignocellulosic biomass representing agricultural residues and herbaceous grasses would be probable. Corn stover, a lignocellulosic biomass, is used in this study. The model can be applicable to such biomasses with similar biochemical compositions within the studied HTL process range of 250–350 °C. However, the application to outside these boundaries should be examined in future work.

## 4 Conclusions

This study developed an element-based kinetic model to predict the yields, HHVs, and fuel characteristics (H/C and O/C ratios) of corn-stover-derived HTL products under operating conditions of 5–15 g of solid loading, 250–350 °C, and 5–60 min. The model reproduced the total weights of SR and HBO with RMSE values of 0.73 and 1.06 g, respectively, and the combined weights of C and N in AP solutes with an RMSE of 0.62 g. The HHVs of SR and HBO were well predicted, with relative error ranges of −30% to 26% for SR and −7% to 18% for HBO; notably, the HBO HHV at 350 °C showed a narrow RE range (−6.6% to 0.1%). Fuel characteristics derived from predicted elemental compositions of SR and HBO were also consistent with experimental data, with RMSE values of 0.061 for SR and 0.060 for HBO. In addition, power function relationships were observed between corn stover weight and the corresponding SR and dissolved organic carbon in AP when solid loading was varied, despite the expectation of linearity under a pseudo-first-order assumption. These results suggest the presence of non-pseudo-first-order pathways, particularly in feedstock conversion to hydrochar, and demonstrate the model’s capability to provide both predictive accuracy and mechanistic insight into HTL reactions.

## ∇

Global Institute of Future Technology, Shanghai Jiao Tong University, 800 Dongchuan Avenue, Shanghai 200240, China

## Abbreviations

HTL: hydrothermal liquefaction
CS: corn stover
HC: hydrochar, SR, solid residue
AP: aqueous phase
HBO: heavy bio-oil
RMSE: root-mean-square error
RE: relative error
ODE: ordinary differential equation
